# U-Shaped Relationship between Serum Leptin Concentration and Cognitive Performance in Older Asian Adults

**DOI:** 10.3390/nu11030660

**Published:** 2019-03-19

**Authors:** Cedric Annweiler, Guillaume T. Duval, Ching-Yu Cheng, Tien-Yin Wong, Ecosse L. Lamoureux, Dan Milea, Charumathi Sabanayagam

**Affiliations:** 1 Department of Geriatric Medicine, Angers University Hospital, University Memory Clinic of Angers, UPRES EA 4638, University of Angers, 49100 Angers, France; Cedric.Annweiler@chu-angers.fr (C.A.); guillaume.duval@chu-angers.fr (G.T.D.); 2 Robarts Research Institute, Department of Medical Biophysics, Schulich School of Medicine and Dentistry, The University of Western Ontario, London, ON N6A 3K7, Canada; 3 Singapore Eye Research Institute, Singapore National Eye Centre, 11 Third Hospital Avenue, #06-13, SNEC Bldg, Singapore 168751, Singapore; chingyu.cheng@duke-nus.edu.sg (C.-Y.C.); wong.tien.yin@singhealth.com.sg (T.-Y.W.); ecosse.lamoureux@seri.com.sg (E.L.L.); dan.milea@snec.com.sg (D.M.); 4 Ophthalmology and Visual Sciences Academic Clinical Program, Duke-NUS Medical School, Singapore 169857, Singapore; 5 Department of Ophthalmology, Angers University Hospital, 49100 Angers, France

**Keywords:** leptin, cognition, older adults

## Abstract

The role of leptin (a hormone related to fat mass) in cognition remains equivocal. Our objective was to investigate the relationship between circulating leptin concentration and cognition in older adults, accounting for potential confounders. We categorized 1061 community-dwelling older participants ≥60 years (mean ± SD, 70.6 ± 6.4 years; 41.6% female) from the Singapore Kidney Eye Study according to quintiles of leptin concentration (≤2.64; 2.64–5.1; 5.2–8.6; 8.7–17.96; ≥18 ng/mL). Cognition was assessed using the total and domain scores of the Abbreviated Mental Test (AMT). Age, gender, body mass index, mean arterial pressure, smoking, alcohol, education, memory complaint, anxiodepressive disorders, circulating concentrations of 25-hydroxyvitamin D, glycosylated hemoglobin, low-density lipoprotein cholesterol, and estimated glomerular filtration rate were used as potential confounders. Participants within the lowest (Q1) and highest (Q5) leptin quintiles exhibited lower (i.e., worse) mean total AMT scores compared to those within the intermediate quintiles (Q2, Q3, and Q4). Compared to Q3 as the reference, Q1 and Q5 were associated with decreased total AMT score (respectively, β = −0.53 *p* = 0.018; β = −0.60 *p* = 0.036). Compared to Q3, Q5 was also associated with decreased subscores on anterograde (β = −0.19 *p* = 0.020) and retrograde episodic memories (β = −0.18 *p* = 0.039). We found a non-linear U-shaped relationship between circulating leptin and cognition, with both lower and higher concentrations of leptin being associated with more severe cognitive impairment in community-dwelling older Asians.

## 1. Introduction

Leptin is a hormone that is mainly secreted in white adipose tissue [1], and its circulating concentration positively correlates with body fat mass [2]. Classically, leptin is recognized to be involved in energy homeostasis, insulin sensibility, and immune and neuroendocrine function [1,3]. However, it has also recently been shown that several additional biological targets are mediated by the leptin receptor, notably in the brain [4,5,6,7], and specifically in the hippocampus [8,9]—a key region for cognition and memory.

A few neuroepidemiological studies have suggested a possible association between serum leptin levels and cognitive performance in older adults [10,11,12,13,14,15,16,17]. Most of these have reported an association between lower serum leptin concentrations and higher risk for Alzheimer disease [4,5,6,7,8,9,10,11]. Lower serum leptin has been associated with a reduction of hippocampal volume, involved in memory [9]. However, other studies have not supported these findings [15,16,17] or even found an inverse relationship between serum leptin and cognitive decline and dementia [13,15]. This possible contradiction between previous results may be explained by insufficient control of potential confounders (e.g., advance in age, gender, adipose mass, and other metabolic markers), or by a plausible non-linear U-shaped relationship. In the latter case, both lower and higher serum leptin concentrations would be associated with cognitive impairment. The aim of the current study was to explore a possible association between circulating leptin concentration and cognition in a population of older community-dwellers in Singapore, while accounting for potential confounders.

## 2. Materials and Methods

### 2.1. Participants

Data for the current analysis were derived from a case–control study designed to examine the association of novel biomarkers including leptin with renal and retinal diseases (Singapore Kidney Eye Study; *n* = 2944) [18]. The study was nested within two independent population-based cross-sectional studies: the Singapore Indian Eye Study (SINDI, 2007–2009), and Singapore Chinese Eye Study (SCES, 2009–2011). Both studies followed the same study protocol, were conducted in the same study clinic (Singapore Eye Research Institute), and recruited Indian and Chinese adults aged 40–80 years residing in the southwestern part of Singapore though an age-stratified random sampling method. Details of the methodology and population characteristics of SINDI, SCES, and the Singapore Kidney Eye Study were published previously [18,19,20]. All participants underwent an interview, as well as clinical and laboratory examinations at the study clinic. For the current analysis, we included only those aged ≥60 years, as cognitive status was assessed only among these participants.

### 2.2. Circulating Leptin Concentration

Circulating leptin concentration was measured from venous blood collected in the non-fasting state at baseline and stored at −80 °C. The collection and storage process was the same for all study participants. Serum samples were used in Chinese and Indian subjects, and plasma samples were used in Malays for measuring leptin. Circulating leptin levels were measured using a commercial ELISA kit (EMD Millipore, MO, USA) at the National University Hospital laboratory. The lower limit of detection of leptin in the current study was 0.78 ng/mL. The sensitivity of the assay was 0.135 ng/mL + 2SD. The coefficient of variation (CV) was below 10% (2.6%–6.2%) at concentrations ranging from 2.34 ng/mL to 28.9 ng/mL. First, the leptin concentration was categorized into ranges of 4 ng/mL to avoid saturated and unreadable scatter plot of the relationship between leptin concentration and cognitive performance. Data were grouped for circulating leptin concentration ≥20 ng/mL, as the number of participants was low. Second, leptin levels were categorized based on quintilization: quintile 1 less than or equal to 2.64 ng/mL, quintile 2 between 2.65 and 5.1 ng/mL, quintile 3 between 5.2 and 8.6 ng/mL, quintile 4 between 8.7 and 17.96 ng/mL, and quintile 5 greater than or equal to 18 ng/mL.

### 2.3. Assessment of Cognitive Performance

Cognition was assessed using the Abbreviated Mental Test (AMT), a reliable standardized and validated screening test for cognitive impairment [21,22]. The AMT is notably sensitive (91.5%) and specific (82.4%) to dementia in adults aged ≥60 years [23]. It consists of 10 items scored 0 or 1 (correct answer). Global cognitive performance was explored with the total AMT score, which ranges from 0 (no correct answer) to 10 (all answers correct). Decreasing AMT score is synonymous with increasing severity of cognitive impairment. Global cognitive impairment was defined as AMT score ≤6 if education 0–6 years, or AMT score ≤8 if education >7 years [19,20]. The number of education years was noted from a standardized questionnaire. In addition, domain-specific cognitive performances were separately analyzed by decoupling AMT items referring to anterograde episodic memory (recall of memory phrase), retrograde episodic memory (date of birth; home address), semantic memory (name prime minister; name picture), working memory (serial subtraction from 20), and orientation in space (place) and in time (hour; year). Each performance was considered impaired if one or more items was not answered correctly.

### 2.4. Assessments of Potential Confounders

The following potential confounders were assessed: age, gender, history of regular smoking and of regular alcohol consumption, education level, body mass index (BMI), mean arterial pressure (MAP), subjective memory complaint, anxiodepressive disorders, circulating concentrations of glycosylated hemoglobin (HbA1c), low-density lipoprotein (LDL) cholesterol and 25-hydroxyvitamin D (25OHD), and estimated glomerular filtration rate (eGFR).

An interviewer-administered standardized questionnaire was used to obtain information on age, gender, subjective memory complaint, history of regular smoking, alcohol consumption, highest education level attained, and mood. High education level was defined as ≥6 education years. Regular alcohol consumption was defined as ≥5 days a week. Anxiodepressive disorders were defined as reporting moderate-to-extreme anxiety or depression. Clinical covariates were obtained from a standardized physical examination. Systolic blood pressure (SBP) and diastolic blood pressure (DBP) were measured using a digital automatic blood pressure monitor after the participant was seated for at least 5 min and an average of two measurements were taken as the blood pressure value for that individual. MAP was calculated as: $\frac{\mathrm{SBP}+2\;\mathrm{DBP}}{3}$. Circulating creatinine, HbA1c (in %), LDL cholesterol (mmol/L), and 25OHD (µg/L) were determined using automated standard laboratory methods at the Singapore General Hospital laboratory. eGFR was estimated in mL/min from standardized serum creatinine using the CKD-EPI equation [24].

### 2.5. Statistical Analysis

Participant characteristics were summarized using means and standard deviations (SDs) or frequencies and percentages, as appropriate. As the number of observations were higher than 40, comparisons were not affected by the shape of the error distribution, and no transform was applied [25]. Firstly, comparisons among participants separated into five groups based on quintiles of circulating leptin concentration were performed using the Chi-square (Chi^2^) test or ANOVA, as appropriate. Further post-hoc analyses were performed using Fisher’s least significant difference test. Secondly, univariate and multiple linear regressions were used to examine the association between cognitive performance (dependent variable) and each quintile of leptin concentration (independent variable), while adjusting for all studied potential confounders and using the intermediate quintile as the reference (i.e., odds ratio = 1). Separate models were used to explain the total AMT score as well as all domain-specific AMT subscores (i.e., anterograde episodic memory, retrograde episodic memory, semantic memory, working memory, orientation in space, and orientation in time). *p*-values < 0.05 were considered significant. All statistics were performed using SPSS (v15.0, IBM Corporation, Chicago, IL, USA).

### 2.6. Ethics

Subjects participating in the study were included after having given their written informed consent for research. The study was conducted in accordance with the ethical standards set forth in the Helsinki Declaration (1983). The SEED and Singapore Kidney and Eye Study were approved by the Institutional Review Boards (SEED: Singapore Ministry of Health’s National Medical Research Council (NMRC) 0796/2003, 1149/2008, STaR/0003/2008, and Biomedical Research Council (BMRC), Singapore 08/1/35/19/550; Singapore Kidney and Eye Study: NMRC/TA/0008/2012).

## 3. Results

A total of 1061 participants were recruited in this analysis (mean ± SD, 70.6 ± 6.4 years; 41.6% female; AMT score = 8.7 ± 1.7). Overall, the mean ± SD measured leptin concentration was 12.1 ± 15.5 ng/mL (Table 1).

**Figure 1:**
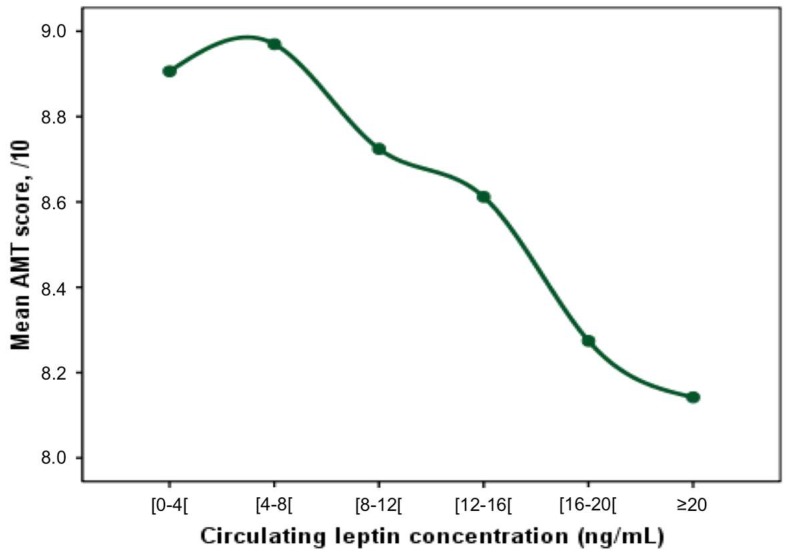
Mean Abbreviated Mental Test score (/10) according to the circulating leptin concentration (ng/mL).

A first visual representation of the relationship between circulating leptin concentration and AMT score showed a U-curve, with a decrease in cognitive performance for low leptin concentrations, but also for high leptin concentrations (Figure 1).

**Figure 2:**
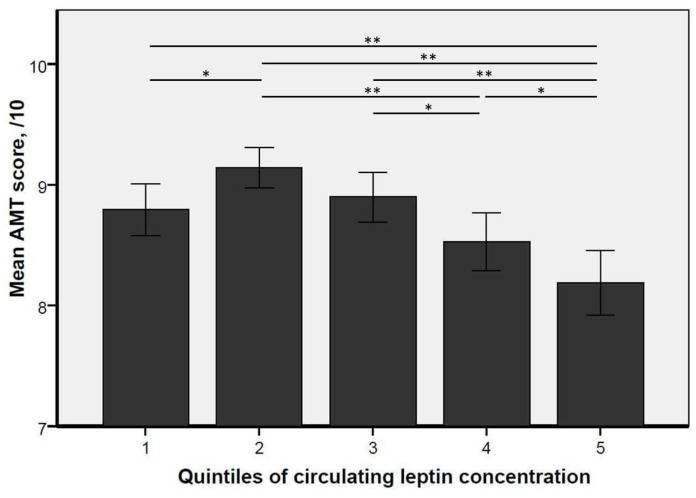
Bar plots for mean and standard deviation of Abbreviated Mental Test score according to the quintiles of circulating leptin concentration (*n* = 1061; overall *p*-value < 0.001). *: *p* < 0.05; **: *p* < 0.001.

As indicated in Table 1, participants categorized according to the quintiles of leptin concentration differed for all covariates except age, alcohol consumption, MAP, memory complaint, and LDL cholesterol concentration. In particular, the proportion of women, the BMI, and the proportion of anxiodepressive disorders increased from the lowest to the highest quintile (*p* for trend <0.001 using Jonckheere–Terpstra test). We also found that participants in Q1 and Q5 had significantly lower mean AMT scores compared to those within the intermediate quintiles (i.e., Q2, Q3, and Q4, Figure 2). The mean ± SD AMT score was 8.8 ± 1.6 ng/mL in Q1 and 8.2 ± 2.0 ng/mL in Q5, although the highest mean AMT score was found for Q2 (9.1 ± 1.2 ng/mL).

Table 2 shows univariate and multiple linear regressions between the quintiles of circulating leptin concentration (independent variable) and the AMT score and subscores (dependent variable). Compared to the intermediate third quintile used as the reference, the lowest and highest quintiles of circulating leptin concentration were associated with a decrease in the total AMT score (fully adjusted β = −0.53 with *p* = 0.018, and fully adjusted β = −0.60 with *p* = 0.036, respectively). With regards to the domain-specific cognitive subscores, we found no difference between the lowest and intermediate quintiles. In contrast, the highest quintile was associated with worse score on anterograde (fully adjusted β = −0.19 with *p* = 0.020) and retrograde episodic memories (fully adjusted β = −0.18 with *p* = 0.039) compared to the third quintile. The other associations were not significant in multivariate models (Table 2).

## 4. Discussion

Our main finding was that, independent of all measured potential confounders, both lower and higher concentrations of leptin were associated with more severe cognitive impairment among community-dwelling older adults, specifically affecting episodic memory. This U-shaped relationship suggests that older adults with low or high circulating leptin concentrations are particularly prone to exhibiting cognitive impairments for which leptin may be a modifiable risk factor.

Few studies with mixed results are available on the association between leptin concentration and cognitive function. Most of the previous ones reported a significant inverse association between leptin concentration and cognitive performance [4,5,6,7,8,9,10,11,12]. Lower leptin concentration was associated with lower (i.e., worse) cognitive scores [4,7,10,11,12]. Other studies did not find any association between leptin status and cognitive performance [15,16,17]. In contrast, other studies have shown a larger frequency of dementia among people with higher leptin concentration [13,14,15,26,27,28]. These mixed findings may be explained by the fact that the relationship between leptin and cognition could be more complex than originally expected, and not linear. Indeed, it has been previously suggested that the impact of leptin on cognitive performance may follow a curvilinear relationship [29].

First, it is possible that, up to a certain threshold, leptin is protective to the brain. The have been experimentation reports that leptin may prevent cortical atrophy [5,13,30], but also amyloid accumulation and hyperphosphorylation of TAU peptides [4,8,31]—two hallmarks of Alzheimer’s disease. It has also been shown that leptin modulates neuronal excitability through glutamate receptors [4,8], and promotes hippocampal synaptic density and synaptic plasticity [4,32]. In addition, leptin may be neuroprotective through an anti-apoptotic effect [4]. Finally, some studies report a cytoprotective effect of leptin through the improvement of mitochondrial function and the prevention of mitochondrial neurotoxins [33]. These neuroprotective effects of leptin on the brain may account for the better cognitive performance seen in our study among participants within the intermediate quintiles of leptin concentration compared to the lowest quintile (Figure 2).

Second, it is likely that such beneficial effects of leptin could be surpassed. For instance, higher serum leptin concentrations are known to accompany increased BMI and metabolic syndrome, which expose individuals to greater cerebrovascular risk. The consequence is that people with overweight and increased leptin status are more exposed to vascular dementia than the others. Moreover, a central resistance to leptin has also been described during obesity [34], which may expose subjects to the same manifestations as the lack of leptin, that is, cognitive impairments (if confirmed). These mechanisms may explain why some previous studies reported a greater prevalence of vascular dementias in the case of increased leptin concentrations [15,26,27,28], together with the present result showing worse cognitive performance in the highest quintile of leptin compared to the intermediate quintiles of leptin (Figure 2)—notably worse episodic memory, a domain-specific cognitive function underpinned by the hippocampus.

The finding of a U-shaped relationship between circulating leptin concentration and cognitive performance has interesting potential implications. To the best of our knowledge, there are no clear reference values for a “normal” leptin status in older adults. In general, there are two ways of establishing reference values for a biological variable. The first one is based on the use of “population-based reference values”, which comprises measuring a parameter in the reference population size and calculating the reference interval involving 95% of the population. The second method is to define abnormal status as the onset of adverse health effects, known as “health-based reference values”. In our study, using population-based reference values would define normality for circulating leptin concentration as a range of concentrations between 0.8 and 55.2 ng/mL. However, it appears clearly from Figure 1 and Figure 2 that such a wide range of concentrations may actually expose older adults to cognitive impairments. Thus, our results encourage the use of health-based reference values for leptin, and to take into account the probable U-shaped relationship between leptin and cognition. From the visual reading of our results, we propose a range between 2.5 and 8.5 ng/mL for reference leptin concentrations in older adults (i.e., Q2 and Q3). Such estimates may help to justify, plan, evaluate, and compare the effectiveness of interventions aiming at preventing cognitive decline that would consider leptin as a modifiable risk factor.

Strengths of our study include the relatively large size of the sample of older participants representing two major ethnic groups in Asia—a geographic area in which the association between leptin and cognition has not yet been studied. Additionally, leptin concentration was measured using a standardized accurate and validated automated technique. Beside global cognitive performance, the use of AMT subscores allowed the assessment of specific aspects of cognition in relation to leptin. Finally, linear regression models were applied to measure adjusted associations.

This study has a number of inherent limitations. First, the study cohort was restricted to community-dwelling Indian and Chinese adults who might not be representative of all older Indian and Chinese adults in Singapore. Second, no information was available on possible history of leptin repletion in studied participants. Third, a selection bias is possible since data were derived from a case–control study designed to examine the association of novel biomarkers including leptin with renal and retinal diseases [18]. This also explains why the BMI, rather than the hip-to-waist ratio, was used here although the hip-to-waist ratio is suggested to be more relevant in the case of neurodegeneration or cognitive impairment. Fourth, limitations include the use of AMT subscores which consisted of one or two items and provided a lower standard of proof compared to memory-dedicated tests but were examined to identify areas that deserve future studies. Fifth, although we were able to control for many characteristics likely to modify the relationship between circulating leptin concentration and cognitive performance, residual potential confounders may still be present, such as the quality and quantity of daily dietary intakes; the ApoE status; or the serum uric acid, triglyceride, and homocysteine levels. Finally, the use of a cross-sectional design prevented any causal inference. In particular, as diet plays a role in the onset of dementia—even if not completely understood at present—it is noticeable that other dietary metabolites, including the serum uric acid, show a U-shaped relationship with cognition that is very similar to the one we found here with leptin [35]. Additionally, recent studies have reported that gut microbiota (i.e., the ensemble of bacteria, fungi, viruses, protozoa, and archaea symbiotically living in the distal human gastrointestinal tract under various external influences, including the diet) may be involved in cognitive decline by promoting chronic inflammation and anabolic resistance [36]. It is thus possible that leptin may actually represent a marker of dietary patterns influencing cognitive function, rather than a mediator by itself.

## 5. Conclusions

In conclusion, we report a likely U-shaped association between circulating leptin concentrations and cognitive performance among community-dwelling older adults in Singapore. This suggests that older adults with both lower and higher circulating leptin concentrations are particularly prone to exhibit cognitive impairment for which leptin may be a modifiable risk factor. These novel findings need to be replicated in larger and preferably longitudinal cohorts with extensive evaluation of the diet, and ideally considering not only the cognitive outcomes but also some neurodegenerative biomarkers such as the TAU and amyloid beta peptides or brain atrophy.

## Figures and Tables

**Table 1:** Participant data.

		Quintiles of Circulating Leptin Concentration, ng/mL					Overall *p*-Value *
	Whole Cohort (*n* = 1061)	Q1 (*n* = 212) ≤2.64	Q2 (*n* = 206) 2.65–5.1	Q3 (*n* = 222) 5.2–8.6	Q4 (*n* = 212) 8.7–17.96	Q5 (*n* = 209) ≥18	
**Demographical measures**							
Age, years	70.6 ± 6.4	71.5 ± 6.5	69.9 ± 6.2	70.7 ± 6.3	70.6 ± 6.6	69.9 ± 6.4	0.067
Female gender, n (%)	441 (41.6)	32 (15.1)	35 (17.0)	77 (34.7)	122 (57.5)	175 (83.7)	**<0.001 ^b,c,d,e,f,g,h,i,j^**
Regular smoking, n (%)	342 (32.2)	98 (46.2)	90 (43.7)	91 (41.0)	44 (20.8)	19 (9.1)	**<0.001 ^c,d,f,g,h,i,j^**
Regular alcohol consumption ^†^, n (%)	16 (1.5)	6 (2.8)	5 (2.4)	2 (0.9)	0 (0.0)	3 (1.4)	0.112
High education level ^‡^, n (%)	447 (42.1)	90 (42.5)	101 (49.0)	102 (45.9)	83 (39.2)	71 (34.0)	**0.019 ^f,g,i^**
**Clinical measures**							
Body mass index, kg/m^2^	24.8 ± 4.6	21.2 ± 2.9	23.5 ± 2.7	24.1 ± 2.9	25.3 ± 3.2	29.8 ± 5.7	**<0.001 ^a,b,c,d,e,f,g,h,i,j^**
Mean arterial pressure, mmHg	98.3 ± 11.0	98.5 ± 11.2	98.2 ± 10.8	98.6 ± 10.8	98.5 ± 11.1	97.5 ± 11.1	0.810
Memory complaint, n (%)	292 (48.6)	69 (50.4)	53 (49.5)	59 (49.2)	60 (50.8)	51 (42.9)	0.730
Anxiodepressive disorders, n (%)	282 (26.6)	35 (16.5)	39 (18.9)	51 (23.0)	68 (32.1)	89 (42.6)	**<0.001 ^c,d,f,g,h,i,j^**
**Blood measures**							
Leptin, ng/mL	12.1 ± 15.5	1.5 ± 0.6	3.7 ± 0.7	6.7 ± 1.0	12.6 ± 2.66	36.4 ± 20.3	-
Glycosylated hemoglobin, %	6.4 ± 1.1	6.2 ± 1.1	6.3 ± 1.1	6.4 ± 1.0	6.5 ± 1.1	6.5 ± 1.0	**0.003 ^b,c,d,f^**
LDL cholesterol concentration, mmol/L	3.0 ± 0.9	3.1 ± 0.9	2.9 ± 0.8	3.0 ± 1.0	3.0 ± 0.9	3.0 ± 0.8	0.249
25-hydroxyvitamin D, µg/L	22.3 ± 11.2	30.1 ± 10.3	27.8 ± 11.1	25.1 ± 10.4	23.1 ± 11.5	20.2 ± 9.8	**<0.001 ^a,b,c,d,e,f,g,h,i,j^**
Estimated glomerular filtration rate, mL/min	69.2 ± 20.3	73.5 ± 17.4	72.4 ± 19.1	69.2 ± 20.4	68.3 ± 20.9	62.5 ± 21.7	**<0.001 ^b,c,d,f,g,i,j^**

Data are presented as mean ± standard deviation where applicable; * Comparisons based on one-way analysis of variance (ANOVA), Fisher’s least significant difference (LSD) test, or Chi-square test, as appropriate; ^†^: ≥5 days a week; ^‡^: ≥ 6 years; ^a^: significant difference between Q1 and Q2; ^b^: significant difference between Q1 and Q3; ^c^: significant difference between Q1 and Q4; ^d^: significant difference between Q1 and Q5; ^e^: significant difference between Q2 and Q3; ^f^: significant difference between Q2 and Q4; ^g^: significant difference between Q2 and Q5; ^h^: significant difference between Q3 and Q4; ^i^: significant difference between Q3 and Q5; ^j^: significant difference between Q4 and Q5; *p*-value significant (i.e., *p* < 0.05) indicated in bold. LDL: low-density lipoprotein.

**Table 2:** Univariate and multiple linear regressions showing the cross-sectional association between the quintiles of circulating leptin concentration (independent variable) and the Abbreviated Mental Test (AMT) score and subscores (dependent variable), adjusted for participants’ characteristics * (*n* = 1061).

	Circulating Leptin Concentration, ng/mL								
	Q1 (Lowest)		Q2		Q3	Q4		Q5 (Highest)	
	β (95% CI)	*p*-Value	β (95% CI)	*p*-Value	β	β (95% CI)	*p*-Value	β (95% CI)	*p*-Value
**AMT score, /10**									
Unadjusted model	−0.10 (−0.40;0.19)	0.489	0.24 (−0.02;0.51)	0.071	**ref**	−0.37 (−0.68;−0.06)	**0.021**	−0.71 (−1.05;−0.38)	**<0.001**
Fully adjusted model *	−0.53 (−0.98;−0.09)	**0.018**	−0.08 (−0.45;0.30)	0.697	**ref**	−0.08 (−0.52;0.36)	0.724	−0.60 (−1.15;−0.04)	**0.036**
**Anterograde episodic memory subscore, /1**									
Unadjusted model	−0.03 (−0.12;0.06)	0.538	0.04 (−0.05;0.13)	0.402	**ref**	−0.06 (−0.16;0.03)	0.192	−0.17 (−0.26;−0.08)	**<0.001**
Fully adjusted model *	−0.06 (−0.21;0.09)	0.409	−0.00 (−0.13;0.13)	0.981	**ref**	−0.28 (−0.16;0.10)	0.677	−0.19 (−0.35;−0.03)	**0.020**
**Retrograde episodic memory subscore, /2**									
Unadjusted model	−0.01 (−0.09;0.07)	0.770	0.06 (−0.01;0.13)	0.094	**ref**	−0.11 (−0.20;−0.02)	**0.018**	−0.19 (−0.28;−0.09)	**<0.001**
Fully adjusted model *	−0.11 (−0.25;0.02)	0.101	−0.01 (−0.12;0.10)	0.824	**ref**	−0.06 (−0.20;0.08)	0.401	−0.18 (−0.35;−0.09)	**0.039**
**Semantic memory subscore, /2**									
Unadjusted model	−0.008 (−0.09;0.08)	0.852	0.04 (−0.05;0.12)	0.404	**ref**	−0.12 (−0.21;−0.03)	**0.012**	−0.12 (−0.22;−0.03)	**0.012**
Fully adjusted model *	−0.05 (−0.18;0.09)	0.504	−0.03 (−0.16;0.10)	0.629	**ref**	−0.05 (−0.19;0.09)	0.450	−0.16 (−0.34;0.01)	0.068
**Working memory subscore, /1**									
Unadjusted model	−0.05 (−0.12;0.02)	0.127	0.03 (−0.03;0.09)	0.357	**ref**	−0.10 (−0.17;−0.03)	**0.006**	−0.10 (−0.17;−0.03)	**0.007**
Fully adjusted model *	−0.10 (−0.21;0.01)	0.074	−0.04 (−0.13;0.06)	0.476	**ref**	−0.06 (−0.16;0.05)	0.279	−0.07 (−0.20;0.06)	0.281
**Orientation in space subscore, /1**									
Unadjusted model	−0.006 (−0.04;0.02)	0.703	0.01 (−0.01;0.04)	0.297	**ref**	0.01 (−0.02;0.03)	0.518	0.00 (−0.02;0.03)	0.807
Fully adjusted model*	−0.05 (−0.11;−0.00)	**0.048**	−0.00 (−0.05;0.04)	0.882	**ref**	0.04 (0.00;0.08)	**0.043**	0.03 (−0.03;0.09)	0.287
**Orientation in time subscore, /2**									
Unadjusted model	0.003 (−0.06;0.07)	0.922	0.06 (0.01;0.12)	**0.031**	**ref**	−0.00 (−0.07;0.07)	0.966	−0.11 (−0.19;−0.04)	**0.004**
Fully adjusted model *	−0.09 (−0.20;0.02)	0.116	0.02 (−0.07;0.11)	0.700	**ref**	0.07 (−0.03;0.17)	0.186	−0.01 (−0.14;0.13)	0.922

β: coefficient of regression corresponding to a change in the cognitive score; CI: confidence interval; Q: quintiles of circulating leptin concentration (i.e., ≤2.64; 2.65–5.1; 5.2–8.6; 8.7–17.96; ≥18 ng/mL); *: Adjusted for age, gender, history of regular smoking and of regular alcohol consumption, education level, body mass index, mean arterial pressure, subjective memory complaint, anxiodepressive disorders, circulating concentrations of glycosylated hemoglobin, low-density lipoprotein cholesterol and 25-hydroxyvitamin D, and estimated glomerular filtration rate; *p*-value significant (i.e., *p* < 0.05) indicated in bold.
